# Tungsten oxide/fullerene-based nanocomposites as electrocatalysts and parasitic reactions inhibitors for VO^2+^/VO_2_^+^ in mixed-acids

**DOI:** 10.1038/s41598-022-18561-6

**Published:** 2022-08-23

**Authors:** Farah A. El Diwany, Taher Al Najjar, Nageh K. Allam, Ehab N. El Sawy

**Affiliations:** 1 Department of Chemistry, School of Sciences and Engineering, The American University in Cairo, New Cairo, 11835 Egypt; 2 Energy Materials Laboratory, School of Sciences and Engineering, The American University in Cairo, New Cairo, 11835 Egypt

**Keywords:** Chemistry, Energy science and technology, Materials science

## Abstract

The relatively high cost of all-vanadium redox flow batteries (VRFBs) limits their widespread deployment. Enhancing the kinetics of the electrochemical reactions is needed to increase the power density and energy efficiency of the VRFB, and hence decrease the kWh cost of VRFBs. In this work, hydrothermally synthesized hydrated tungsten oxide (HWO) nanoparticles, C_76_, and C_76_/HWO were deposited on carbon cloth electrodes and tested as electrocatalysts for the VO^2+^/VO_2_^+^ redox reactions. Field Emission Scanning Electron Microscopy (FESEM), energy-dispersive X-ray spectroscopy (EDX), high-resolution transmission electron microscope (HR-TEM,), X-ray diffraction (XRD), X-ray photoelectron spectroscopy (XPS), Fourier transform infrared spectroscopy (FTIR), and contact angle measurements were used to characterize the electrodes’ material. The addition of the C_76_ fullerene to HWO was found to boost the electrode kinetics towards the VO^2+^/VO_2_^+^ redox reaction, by enhancing the conductivity and providing oxygenated functional groups at its surface. A composite of HWO/C_76_ (50 wt% C_76_) was found to be the optimum for the VO^2+^/VO_2_^+^ reaction, showing a ΔE_p_ of 176 mV, compared to 365 mV in the case of untreated carbon cloth (UCC). Besides, HWO/C_76_ composites showed a significant inhibition effect for the parasitic chlorine evolution reaction due to the W-OH functional groups.

## Introduction

The intensive human activities and the rapid industrial revolution have led to unstoppable high demand for electrical energy that increases yearly by about 3%^1^. The extensive use of fossil fuels as an energy source for several decades resulted in greenhouse gas emissions that have contributed to global warming, water pollution, and air pollution, threatening the entire ecosystem. Therefore, the penetration of clean and renewable wind and solar energies is expected to reach up to 75% of the total electrical energy by 2050^1^. However, the electrical grid becomes unstable when the power from renewable energy sources exceeds 20% of the total generated power^1^. Developing efficient energy storage systems is crucial for such a transition since they are required to store surplus electricity and balance supply and demand.

Among all energy storage systems such as hybrid vanadium redox flow batteries^2^, all-vanadium redox flow batteries (VRFBs) are the most developed for their numerous advantages^3^, and are thought to be an optimal solution for long-term energy storage (~ 30 years) when combined with renewable energy sources^4^. This is due to the decoupling of power and energy densities, fast response, long cycle life, and relatively low annualized cost of $65/kWh in comparison to $93–$140/kWh and $279–$420/kWh for Li-ion and lead-acid batteries, respectively^4^.

However, their extensive commercialization is still impeded by their relatively high system capital cost, primarily due to the cell stack^4,5^. Therefore, improving the cell stack performance by increasing the kinetics of both half-cell reactions can reduce the size of the stack and, consequently, the cost. Therefore, fast electron transfer at the electrode’s surface is needed, which depends on the electrode’s design, composition, and structure that needs to be optimized carefully^6^. Even though carbon-based electrodes have good chemical and electrochemical stability and good conductivity, with no treatment they suffer from sluggish kinetics due to the lack of oxygen functional groups and hydrophilicity^7,8^. Therefore, different electrocatalysts were incorporated with the carbon-based electrodes, specifically carbon nanostructures and metal oxides, to enhance the kinetics at both electrodes to increase the kinetics at the VRFB electrodes.

Many carbon materials have been used, such as carbon paper^9^, carbon nanotubes^10–13^, graphene-based nanostructures^14–17^, carbon nanofibers^18^, and other^19–23^, except the fullerene family. In our previous work on C_76_, we reported for the first time, the superior electrocatalytic activity of this fullerene towards VO^2+^/VO_2_^+^, showing a 99.5% and 97% decrease in charge transfer resistance in comparison to thermally treated and untreated carbon cloth^24^. A summary of the carbon materials' catalytic performance towards the VO^2+^/VO_2_^+^ reactions in comparison to C_76_ is given in Table S1. On the other hand, many metal oxides were used, such as CeO_2_^25^, ZrO_2_^26^, MoO_3_^27^, NiO^28^, SnO_2_^29^, Cr_2_O_3_^30^ and WO_3_^31–38^, owing to their enhanced wettability and abundant oxygen functional groups. A summary of the catalytic performance of these metal oxides towards VO^2+^/VO_2_^+^ reactions is given in Table S2. Quite a few papers used WO_3_ due to its low cost, high stability in acidic media, and high catalytic activity^31–38^. However, the WO_3_ showed an insignificant improvement in the kinetics of the positive electrode. To improve the conductivity of WO_3_, the effect of using the reduced tungsten oxide (W_18_O_49_) on the activity of the positive electrode was tested^38^. The hydrated tungsten oxide (HWO) has never been tested in the VRFB application, despite showing enhanced activity in the supercapacitor application due to faster cation diffusion, compared to the anhydrous WO_x_^39,40^. The third generation of vanadium redox flow batteries uses mixed acid electrolyte composed of HCl and H_2_SO_4_ to enhance the performance of the batters and increase the solubility and stability of vanadium ions in the electrolyte. However, the parasitic chlorine evolution reaction become one of the drawbacks of the third generation and hence finding a way to suppress the chlorine evaluation reaction became the concern of several research groups^41^.

Herein, HWO/C_76_ composites, deposited on carbon cloth electrodes, were tested for the VO^2+^/VO_2_^+^ reaction, aiming at finding a balance between the composites' conductivity and the redox reaction kinetics at the electrode surface, in addition to inhibiting the parasitic chlorine evolution reaction (CER). Hydrated tungsten oxide (HWO) nanoparticles were synthesized via facile hydrothermal methods. Experiments were done in the mixed acid electrolyte (H_2_SO_4_/HCl) to imitate the 3rd Generation (G3) VRFB for more practicality and to study the effect of HWO on the chlorine evolution parasitic reaction^42^.

## Experimental

### Materials

Vanadium (IV) sulfate oxide hydrate (VOSO_4_, 99.9%, Alfa-Aeser), sulfuric acid (H_2_SO_4_), hydrochloric acid (HCl), dimethylformamide (DMF, Sigma-Aldrich), polyvinylidene fluoride (PVDF, Sigma-Aldrich), sodium tungsten oxide dihydrate (Na_2_WO_4_, 99%, Sigma-Aldrich), and ELAT Hydrophilic Plain Carbon Cloth (Fuel Cell Store), were used in this study.

### Synthesis of HWO nanoparticles

Hydrated tungsten oxide (HWO) was fabricated by a hydrothermal reaction^43^, in which 2 g of Na_2_WO_4_ salt was dissolved in 12 mL H_2_O, giving a colorless solution, then 12 mL of 2 M HCl was added dropwise, resulting in a pale-yellow suspension. This suspension was placed in a Teflon-lined stainless autoclave in an oven at 180 ºC for 3 h to undergo the hydrothermal reaction. The residue was collected by filtration, washed three times with ethanol and H_2_O, dried in the oven at 70 ºC for ~ 3 h, and then ground to obtain the grey-blue powder of HWO.

### Preparation of electrodes

The as-received (untreated) carbon cloth (UCC) electrodes were used as it is or thermally treated in a tube furnace in the air at 450 °C for 10 h at a heating rate of 15 ºC/min to produce treated CC (TCC), as in the previous paper^24^. UCC and TCC were cut into ~ 1.5 cm width × 7 cm length electrodes. C_76_, HWO, HWO-10% C_76_, HWO-30% C_76_, and HWO-50% C_76_ suspensions were prepared by adding 20 mg of the active material powder and 10 wt% (~ 2.22 mg) PVDF binder into ~ 1 mL DMF and sonicating for 1 h to enhance homogeneity. 2 mg of C_76_, HWO, and HWO-C_76_ composites was subsequently loaded onto an active electrode area of ~ 1.5 cm^2^ of UCC. All the catalysts were loaded on UCC electrodes and TCC was only used for comparison since it was shown in our previous work that thermal treatment is not necessary^24^. Casting deposition was achieved by brushing 100 µL of the suspension (2 mg loading) for more homogeneity. All electrodes were then oven-dried at 60 °C overnight. Electrodes were measured before and after to ensure accurate mass loading. To have a definite geometric area (~ 1.5 cm^2^) and to prevent the vanadium electrolyte from going up the electrode by capillary effect, a thin layer of wax was applied on top of the active material.

### Physicochemical characterization

Field Emission Scanning Electron Microscopy (FESEM, Zeiss SEM Ultra 60, 5 kV) was used to observe the surface morphology of the HWO. Energy-Dispersive X-ray spectroscopy equipped within the Feii8SEM (EDX, Zeiss Inc.) was used for elemental mapping of HWO-50%C_76_ on UCC electrodes. A high-resolution transmission electron microscope (HR-TEM, JOEL JEM-2100), operating at an accelerating voltage of 200 kV, was used to provide higher resolution imaging and diffraction ring pattern of HWO particles. Crystallographic Tool Box (CrysTBox) software was used to analyze the diffraction ring pattern of HWO using the ringGUI feature, and the results were compared with the XRD pattern^44^. The structure and graphitization of UCC and TCC were analyzed by X-ray diffraction (XRD) at a scan rate of 2.4°/min from 5° to 70° with Cu K_α_ (λ = 1.54060 Å), using Panalytical X-ray diffractometer (model 3600). The crystal structure and phase of HWO were revealed by XRD. PANalytical X’Pert HighScore software was utilized to match the peaks of HWO with the tungsten oxide cards available in the database^45^. Results for HWO were compared with TEM results. The chemical composition and state of the HWO samples were determined by X-ray photoelectron spectroscopy (XPS, ESCALAB 250Xi, ThermoScientific). CASA-XPS software (*v* 2.3.15) was used to deconvolute the peaks and data analysis. Fourier transform infrared spectroscopy (FTIR, Perkin Elmer spectrometer using KBr FTIR grade) measurements were carried out to determine the surface functional groups of HWO and HWO-50%C_76_. Results were compared with XPS results. Contact angle measurements (KRUSS DSA25) were also used to characterize the wettability of the electrodes.

### Electrochemical characterization

A Biologic SP 300 workstation was employed for all electrochemical measurements. Cyclic voltammetry (CV) and electrochemical impedance spectroscopy (EIS) were used to study the electrode kinetics of the VO^2+^/VO_2_^+^ redox reaction and the effect of the reactant (VOSO_4_ (VO^2+^)) diffusion on the reaction rate. A three-electrode cell was used for both techniques with an electrolyte concentration of 0.1 M VOSO_4_ (V^4+^) in 1 M H_2_SO_4_ + 1 M HCl (mixed acid). All the reported electrochemical data was IR corrected. A saturated calomel electrode (SCE) and a platinum (Pt) coil were used as the reference and counter electrodes, respectively. For CV, scan rates (ν) of 5, 20, and 50 mV/s were applied at a potential window of (0–1) V vs. SCE for VO^2+^/VO_2_^+^ and then corrected to the SHE scale for figures plotting (V_SCE_ = 0.242 V vs V_SHE_). To investigate the activity retention of the electrodes, repetitive cycling CVs were conducted at ν of 5 mV/s for UCC, TCC, UCC-C_76_, UCC-HWO, and UCC-HWO-50% C_76_. For EIS measurements, a frequency range of 0.01–10^5^ Hz with a voltage perturbation of 10 mV at the open-circuit voltage (OCV) was used for the VO^2+^/VO_2_^+^ redox reaction. Each experiment was repeated 2–3 times to ensure consistency of the results. The heterogeneous rate constants (k^0^) were obtained by Nicholson’s method^46,47^.

## Results and discussion

### Characterization of HWO and C_76_-HWO nanoparticles

**Figure 1:**
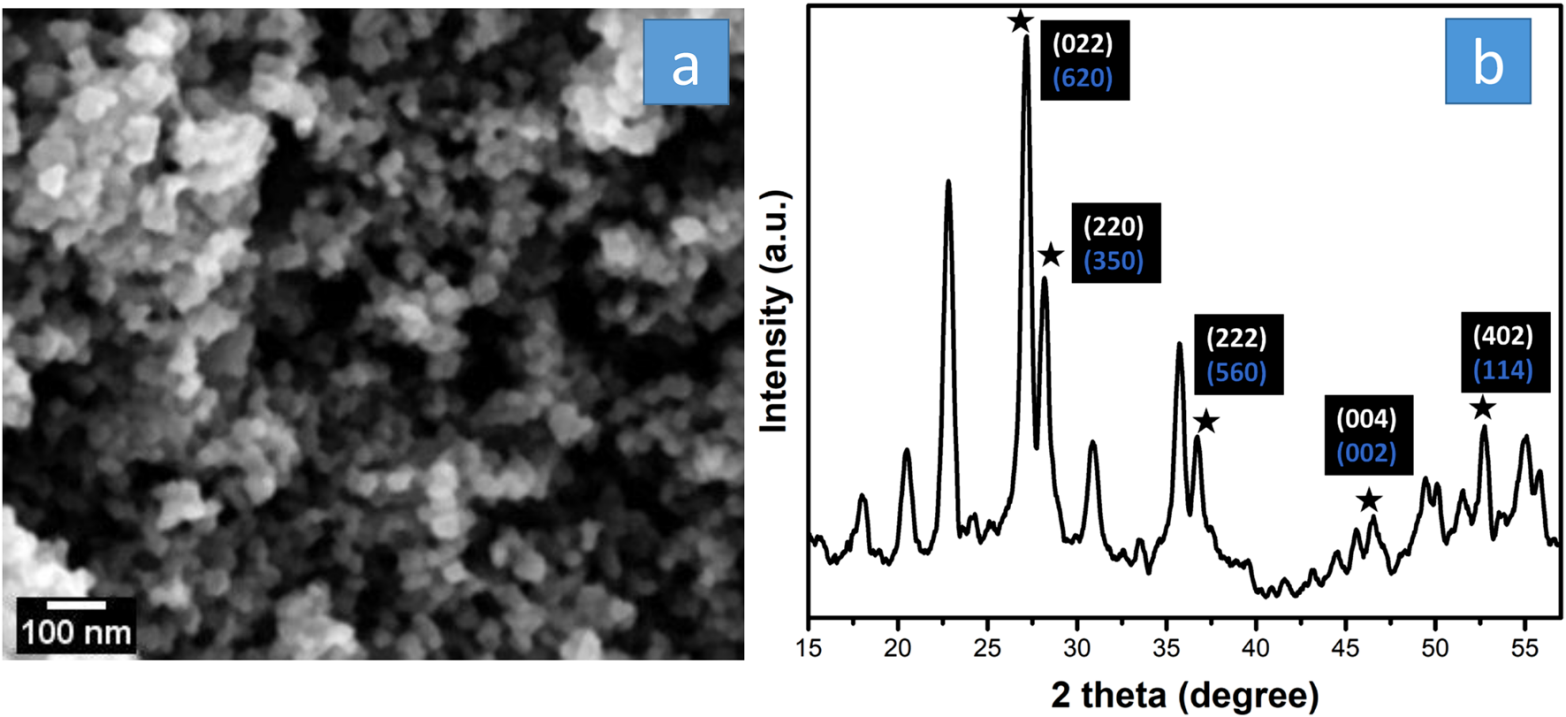
The (**a**) SEM image and (**b**) XRD spectrum of HWO with all diffraction planes.

Hydrated tungsten oxide (HWO) was successfully synthesized via the hydrothermal method. The SEM image in Fig. 1a showed the deposited HWO to consist of clusters of nanoparticles with particle sizes in the range of 25–50 nm.

The XRD pattern of the HWO exhibited (001) and (002) peaks at ~ 23.5° and ~ 47.5°, respectively, characteristic of the non-stoichiometric WO_2.63_ (W_32_O_84_) (PDF 077–0810, a = 21.4 Å, b = 17.8 Å, c = 3.8 Å, α = β = γ = 90°) consistent with their apparent blue color (Fig. 1b)^48,49^. Other peaks at about 20.5°, 27.1°, 28.1°, 30.8°, 35.7°, 36.7°, and 52.7° are allocated to (140), (620), (350), (720), (740), (560) and (970) diffraction planes, of orthorhombic WO_2.63_, respectively^49^. Songara et al.^43^, using the same synthesis method, obtained a white color product, which is related to the existence of WO_3_(H_2_O)_0.333_. However, in this work, a blue-grey color product was obtained due to the different conditions, indicating the coexistence of both the WO_3_(H_2_O)_0.333_ (PDF 087-1203, a = 7.3 Å, b = 12.5 Å, c = 7.7 Å, α = β = γ = 90°) and the reduced form of tungsten oxide. The semi-quantitative analysis done by the X’Pert HighScore software displayed 26% WO_3_(H_2_O)_0.333_: 74% W_32_O_84_. Since the W_32_O_84_ consists of W^6+^ and W^4+^ (1.67:1 of W^6+^:W^4+^), the estimated content of W^6+^ and W^4+^ are about 72% W^6+^ and 28% W^4+^, respectively. SEM image, 1 s core-level XPS spectrum, TEM image, FTIR, and Raman spectra of C_76_ particles are reported in our previous paper^24^. According to Kawada et al.^50,51^, the XRD pattern of C_76_, after toluene removal, showed monoclinic FCC structure.

**Figure 2:**
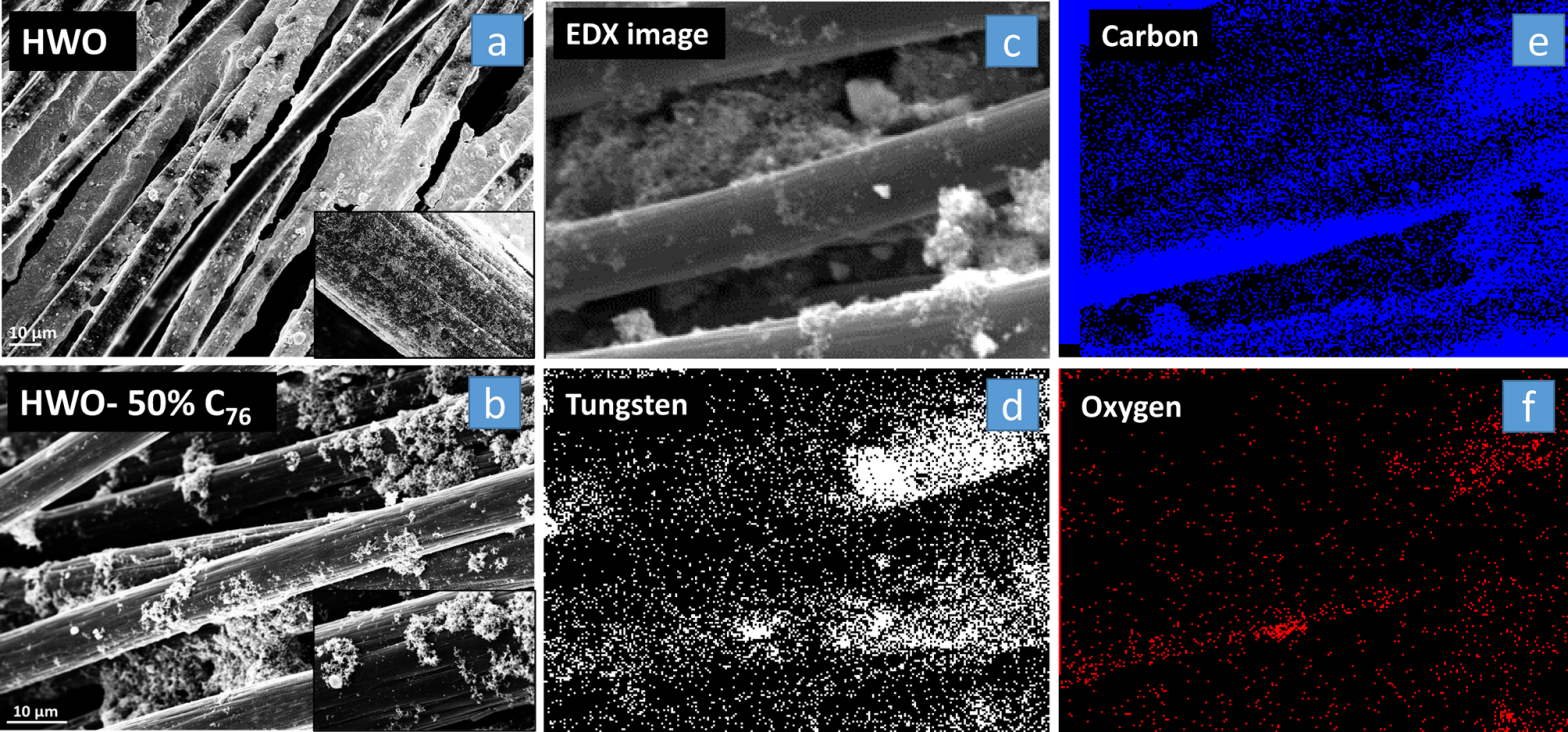
SEM images of the (**a**) deposited HWO and (**b**) HWO-C_76_ particles. EDX mapping on the HWO-C_76_ loaded on UCC using the area in image (**c**) shows the distribution of tungsten (**d**), carbon (**e**), and oxygen (**f**) in the sample.

The SEM images in Fig. 2a and b show the successful deposition of HWO and HWO-50%C_76_ on and between the carbon fibers of UCC electrodes. Tungsten, carbon, and oxygen EDX elemental mapping for the SEM image in Fig. 2c that is demonstrated in Fig. 2d–f show that tungsten and carbon are uniformly mixed (showing similar distribution) all over the electrode surface, with the composite being nonuniformly deposited due to the nature of the deposition method.

**Figure 3:**
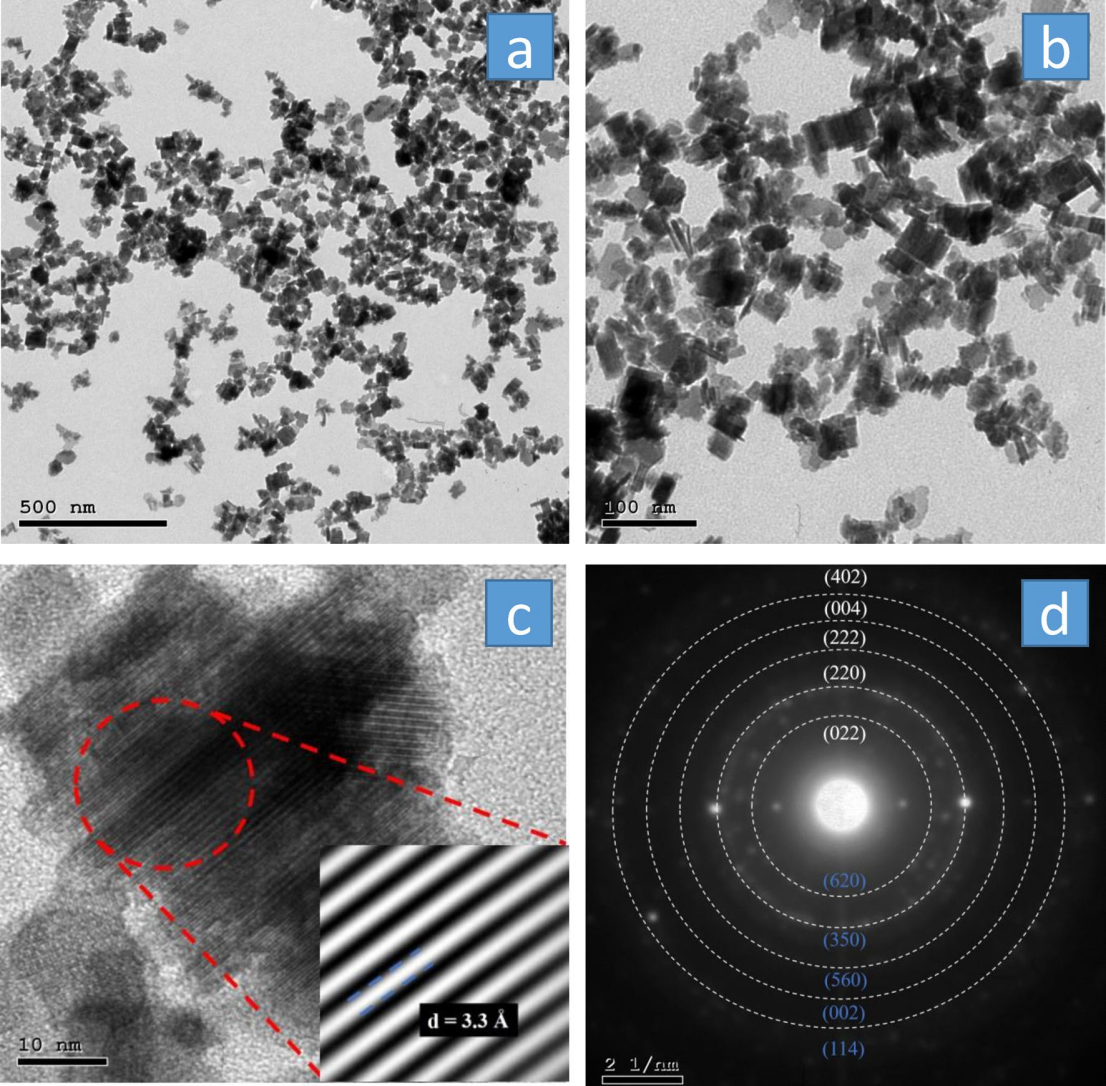
(**a**) The HR-TEM image of the HWO, with (**b**) showing a magnified image. An image of the lattice planes is shown in (**c**), with the inset of (**c**) showing a magnified image of the planes and a d-spacing of 0.33 nm, corresponding to (002) and (620) planes. (**d**) The ring pattern of the HWO, showing the planes associated with WO_3_(H_2_O)_0.333_ (white) and W_32_O_84_ (blue) phases.

HR-TEM was employed for high magnification imaging and crystallography information (Fig. 3). The HWO showed a nanocube morphology as can be seen in Fig. 3a and more clearly in Fig. 3b. Zooming onto the nanocubes for selected area diffraction, the lattice structure, and the diffraction planes satisfying Bragg’s law could be visualized as depicted in Fig. 3c, confirming the crystallinity of the material. The inset of Fig. 3c shows a d-spacing of 3.3 Å, corresponding to the (022) and (620) diffraction planes found in WO_3_(H_2_O)_0.333_ and W_32_O_84_ phases, respectively^43,44,49^. This is consistent with the above XRD analysis (Fig. 1b), as the d-spacing of the observed lattice plane (Fig. 3c) corresponds to the most intense XRD peak in the HWO sample. The ring pattern is also displayed in Fig. 3d with each ring corresponding to a different plane. WO_3_(H_2_O)_0.333_ and W_32_O_84_ planes are written in white and blue, respectively, with their respective XRD peaks also depicted in Fig. 1b. The first ring shown in the ring pattern corresponds to the first marked peak in the XRD pattern of the diffraction plane (022) or (620). From (022) to (402) rings, the d-spacing values were found to be 3.30, 3.17, 2.38, 1.93, and 1.69 Å, which are consistent with the XRD values of 3.30, 3.17, 2.45, 1.93, and 1.66 Å, respectively^44,45^.

**Figure 4:**
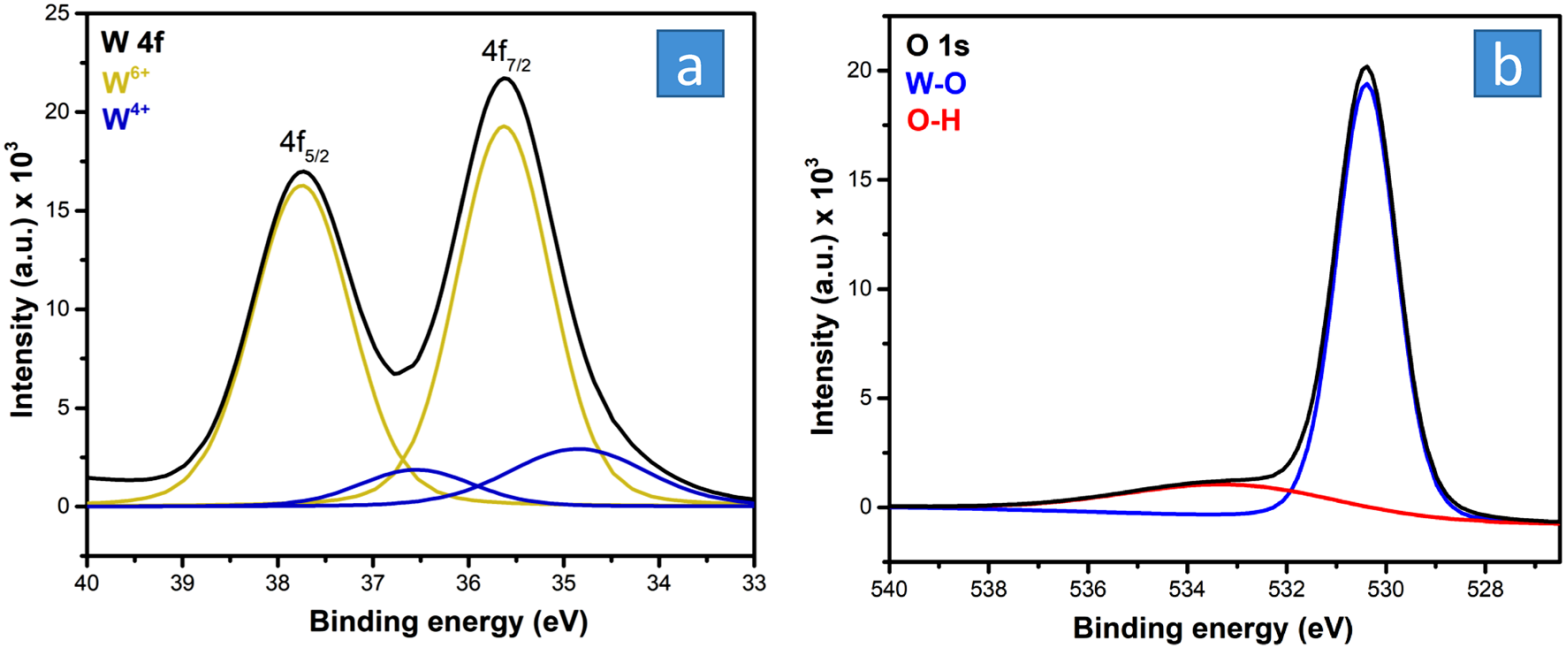
XPS narrow scan spectrum of (**a**) W_4f._ and (**b**) O_1s_ for the synthesized HWO.

The XPS analysis was conducted to determine the surface chemistry and oxidation state of tungsten (Figs. S1 and 4). The wide-range XPS scan spectrum of the synthesized HWO is shown in Fig. S1, indicating the presence of tungsten. The XPS narrow scan spectra of W 4f and O 1s core levels are shown in Fig. 4a and b, respectively. The W 4f spectrum was separated into two spin–orbit doublet peaks corresponding to the binding energies of W oxidation states. Whereas the W 4f_5/2_ and W 4f_7/2_ peaks located at binding energies of 37.8 and 35.6 eV are ascribed to W^6+^, the W 4f_5/2_ and W 4f_7/2_ peaks at 36.6 and 34.9 eV are characteristic of the W^4+^ state, respectively ^40^. The presence of a lower oxidation state (W^4+^) further confirms the formation of non-stoichiometric WO_2.63_, while the presence of W^6+^ indicates stoichiometric WO_3_ attributed to WO_3_(H_2_O)_0.333_. The fitting data reveal that the atomic percentages of W^6+^ and W^4+^ are 85% and 15%, respectively, which are relatively close to the values estimated from the XRD data, taking into consideration the differences between the two techniques. Both techniques give quantitative information with low accuracy, especially XRD. In addition, the two techniques analyze a different portion of materials, as XRD is a bulk technique while XPS is a surface technique that approaches only a few nanometers. The O 1s spectrum was deconvoluted into two peaks at 533 (22.2%) and 530.4 eV (77.8%). The former corresponds to O–H, while the latter is ascribed to the crystal lattice oxygen bond in W–O. The presence of the O–H functional group is consistent with the hydrated nature of HWO.

**Figure 5:**
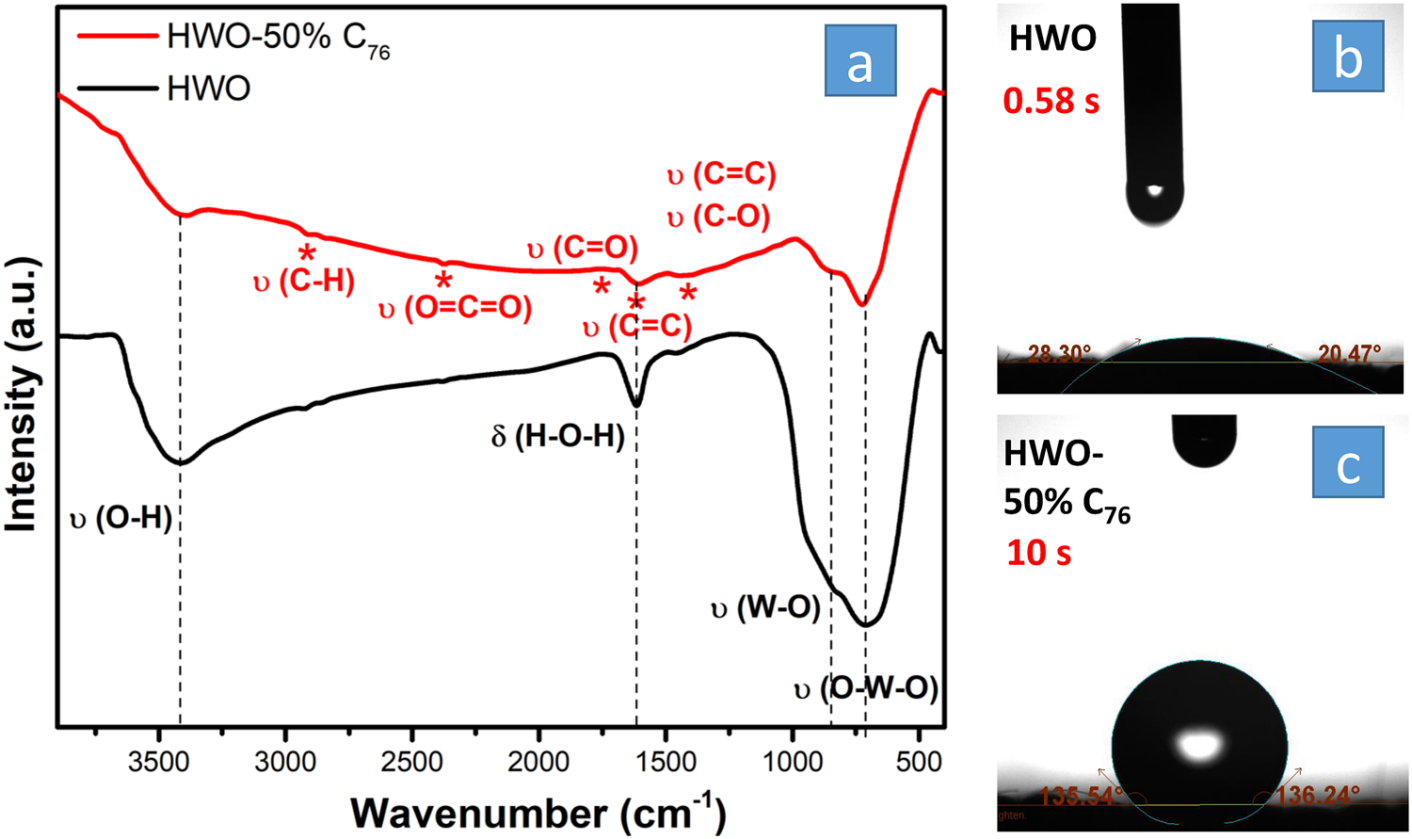
FTIR analysis (**a**) of HWO and HWO-50% C_76_, indicating the functional groups, and the contact angle measurements (**b**, **c**).

The FTIR analysis was also conducted for these two samples to study the presence of functional groups and the coordinated water molecules in the hydrated HWO structure. The results show that the HWO-50% C_76_ sample and HWO FT-IR results seem similar due to the presence of HWO, but with different peak intensities due to the different amount of sample that was used during preparation for analysis (Fig. 5a). The HWO-50% C_76_ show all the peaks reported to fullerene^24^ in addition to that for the tungsten oxide peaks. In detail, Fig. 5a shows that both samples exhibited very strong broadband at ~ 710/cm assigned to the O–W–O stretch vibration in the HWO lattice structure, an intense shoulder at ~ 840/cm attributed to the W–O stretch vibration, a sharp band at ~ 1610/cm referring to the O–H bending vibration, and a broad absorption band at ~ 3400/cm assigned to the O–H stretch vibration in hydroxyl groups^43^. These results are consistent with the XPS spectra in Fig. 4b, where W–O functional groups could provide active sites for the VO^2+^/VO_2_^+^ reaction.

The O–H groups could also catalyze the VO^2+^/VO_2_^+^ reaction, along with increasing the hydrophilicity of the electrode, hence facilitating diffusion and electron transfer rate. The HWO-50% C_76_ sample exhibited additional peaks for C_76_, as marked on the graph. The peaks at ~ 2905, 2375, 1705, 1607, and 1445/cm could be assigned to C–H, O=C=O, C=O, C=C, and C–O stretching vibrations, respectively^14^. As already known, C=O and C–O oxygen functional groups could act as active sites for vanadium redox reactions. To test and compare the wettability of both electrodes, contact angle measurements were employed as shown in Fig. 5b, c. The HWO electrode immediately absorbed the water droplet, indicating super hydrophilicity because of the available O–H functional groups. The HWO-50% C_76_ was more hydrophobic with a contact angle of ~ 135° after 10 s. However, in the electrochemical measurements, the HWO-50% C_76_ electrode became completely wet in less than a minute. The wettability measurements are consistent with the XPS and FTIR results that showed the more abundant O–H groups on the surface of HWO made it relatively more hydrophilic.

### Electrochemical activity of HWO nanoparticles and HWO-C_76_ nanocomposites

HWO and HWO-C_76_ nanocomposites were tested for the VO^2+^/VO_2_^+^ reaction, with the anticipation that HWO would inhibit the chlorine evolution that takes place during the VO^2+^/VO_2_^+^ reaction in mixed acid and the C_76_ to further catalyze the desired VO^2+^/VO_2_^+^ redox reaction. HWO suspensions with 10%, 30%, and 50% C_76_ were prepared and deposited on UCC electrodes with a total loading of ~ 2 mg/cm^2^.

**Figure 6:**
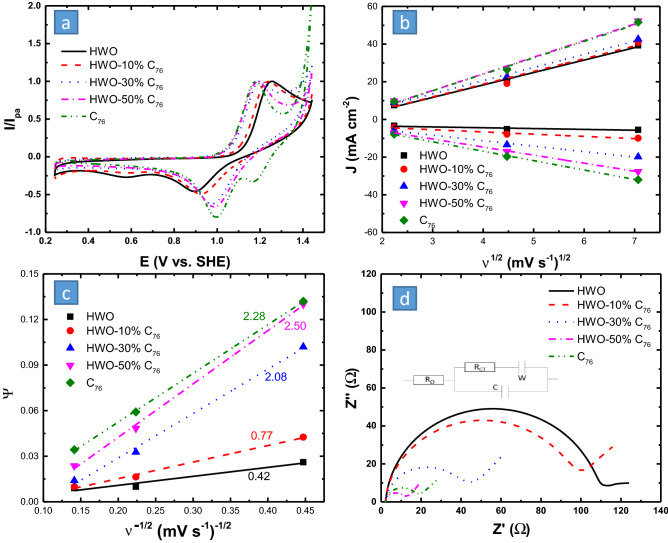
(**a**) The cyclic voltammetry behavior (at ν = 5 mV/s) of the UCC and HWO-C_76_ composites with different HWO:C_76_ ratios for VO^2+^/VO_2_^+^ reactions in 0.1 M VOSO_4_/1 M H_2_SO_4_ + 1 M HCl electrolyte. (**b**) Randles–Sevcik and (**c**) Nicholson’s methods for VO^2+^/VO_2_^+^, to estimate the diffusion efficiency and obtain the k^0^ values (**d**).

The reaction kinetics of VO^2+^/VO_2_^+^ at the electrode surface was examined using CV in the mixed acid electrolyte as shown in Fig. 6. The currents were displayed as *I*/*I*_*pa*_ to make an easier comparison of the *ΔE*_*p*_ and the I_pa_/I_pc_ between the different catalysts directly from the figure. The current per area data is shown in Fig. 2S. Figure 6a shows that the HWO slightly enhances the electron transfer rate of the VO^2+^/VO_2_^+^ redox reaction at the electrode surface and inhibits the parasitic chlorine evolution reaction. While C_76_ significantly enhances the electron transfer rate and catalyzes the chlorine evolution reaction. Therefore, a composite of the HWO and C_76_ with the right composition is expected to have the best activity and the highest ability to suppress the chlorine evolution reaction. Upon increasing the C_76_ content, the electrochemical activity of the electrode was found to improve, as indicated by the reduced ΔE_p_ and increased I_pa_/I_pc_ ratio (Table S3). This was also confirmed by the R_CT_ values (Table S3) extracted from the Nyquist plot in Fig. 6d, which were found to decrease as the C_76_ content increased. These results were also consistent with Lee’s study in which adding mesoporous carbon to mesoporous WO_3_ displayed improved charge transfer kinetics towards VO^2+^/VO_2_^+^^35^. This unraveled the fact that the positive reaction might be much more dependent on the conductivity (C=C bonds) of the electrode^18,24,35–37^. It could also be that C_76_ lowered the overpotential of the reaction by decreasing the organization energy due to the change in coordination geometry between [VO(H_2_O)_5_]^2+^ and [VO_2_(H_2_O)_4_]^+^. This, however, might not be achieved with the HWO electrode.

HWO-50% C_76_ was not only found to exhibit nearly the same electrocatalytic activity of C_76_ towards the VO^2+^/VO_2_^+^ reaction but more interestingly further inhibited the chlorine evolution, relative to C_76_, as shown in Fig. 6a, in addition to displaying a smaller semicircle (lower R_CT_) in Fig. 6d. The C_76_ showed a higher apparent I_pa_/I_pc_ ratio than HWO-50% C_76_ (Table S3), not due to the improvement in the reaction reversibility but due to the overlap with the chlorine reduction reaction peak at 1.2 V vs. SHE. The optimum performance of HWO-50% C_76_ is attributed to the synergistic effect between the negatively charged, highly conductive C_76_ and the high wettability and W-OH catalyzing functional group on HWO. Whereas less chlorine evolution would enhance the charge efficiency of the full cell, the enhanced kinetics would boost the full cell voltage efficiency.

According to Equation S1, for quasi-reversible (relatively sluggish electron transfer) diffusion-controlled reactions, the peak current (I_P_) depends on the number of electrons (n), the electrode area (A), the diffusion coefficient (D), the electron transfer coefficient (α), and scan rate (ν). To check the diffusion-controlled behavior of the tested material, the relation between I_P_ versus ν^1/2^ was plotted and presented in Fig. 6b. Since all materials showed a linear relation the reaction is diffusion-controlled. Since the VO^2+^/VO_2_^+^ reaction is quasi-reversible, the slope of the lines depends on both the diffusion coefficient and the α value (Equation S1). Since the diffusion coefficient is constant (≈ 4 × 10^–6^ cm^2^/s)^52^, the difference in the lines' slop is a direct indication of different α values and hence different electron transfer rates at the electrode surface, with the C_76_ and HWO-50%C_76_ showing the steepest slop (the highest electron transfer rate).

The calculated Warburg (W) slopes, at low frequencies, (Fig. 6d) that are reported in Table S3, have values close to one for all the materials, indicating the ideal diffusion of the redox species and confirming the linear behavior of the I_P_ versus ν^1/2^ of the CV measurements. For the HWO-50% C_76_, the Warburg slope deviated from unity to1.32, indicating the contribution of not only the semi-infinite diffusion of the reactants (VO^2+^) but possibly also a thin layer behavior to the diffusion behavior, due to the electrode porosity.

To further analyze the reversibility (electron transfer rate) of the VO^2+^/VO_2_^+^ redox reaction, Nicholson’s method for quasi-reversible reactions was also used to determine the standard rate constant, k^0^^41,42^. This was done by plotting a dimensionless kinetic parameter, Ψ, which is a function of ΔE_p_, against ν^−1/2^ using equations S2. Table S4 shows the values of Ψ obtained for each electrode material. The results were plotted (Fig. 6c) to obtain k^0^ × 10^4^ cm/s (written next to each line and reported in Table S4) from the slope of each graph using Equation S3. The HWO-50% C_76_ was found to exhibit the highest slope (Fig. 6c), hence the highest k^0^ of 2.47 × 10^–4^ cm/s. This implied that this electrode achieved the fastest kinetics, in line with the CV and EIS results in Fig. 6a and d and Table S3. Besides, k^0^ values were also obtained from the Nyquist plots (Fig. 6d) using R_CT_ values (Table S3) via Equation S4. These results of k^0^ from EIS are summarized in Table S4, and also show that HWO-50% C_76_ exhibited the highest electron transfer rate, owing to the synergistic effects. Even though k^0^ values were not the same due to the different derivations of each method, they still displayed the same order of magnitude and showed consistency.

**Figure 7:**
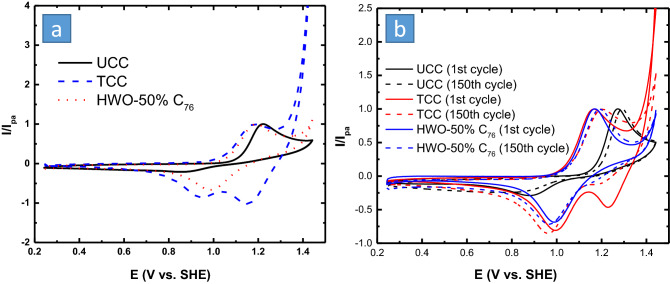
A comparison between the CV of the best electrode materials relative to UCC (**a**) and stability (**b**) for VO^2+^/VO_2_^+^ redox reaction. All CVs were at ν = 5 mV/s in 0.1 M VOSO_4_/1 M H_2_SO_4_ + 1 M HCl electrolyte.

To have a complete picture of the achieved superior kinetics, it was important to compare the best electrode material with bare UCC and TCC electrodes. For the VO^2+^/VO_2_^+^ reaction, HWO-C_76_ did not only display the lowest ΔE_p_ and the best reversibility, but also a significant inhibition of the chlorine evolution parasitic reaction relative to TCC, as seen by the significant drop of current at 1.45 V vs. SHE (Fig. 7a). Regarding the stability, since the catalyst was blended with PVDF binder, and then loaded on the carbon cloth electrode, we assumed that the HWO-50% C_76_ is physically stable. HWO-50% C_76_ showed a peak shift equal to 44 mV after 150 cycles (degradation rate 0.29 mV/cycle), in comparison with 50 mV for UCC (Fig. 7b). This might not be a massive difference, but the kinetics of the UCC electrode was very sluggish and tend to get worse with cycling, especially for the backward reaction. Even though the reversibility of TCC was much better than that of UCC, TCC was found to have a large peak shift of 73 mV after 150 cycles, which could be attributed to a large amount of chlorine evolving on its surface. to make sure that the catalyst will adhere well to the electrode surface. As could be seen for all the tested electrodes, even those with no loaded catalyst, showed cyclic instability to a different extent, indicating that the change in peak separation with cycling is due to the deactivation of the material due to chemical changes, not the catalyst detachment. Furthermore, if a significant amount of the catalyst particles was detached from the electrode surface, it would cause a significant increase in the peak separation (not only 44 mV) since the substrate (UCC) is relatively inactive toward VO^2+^/VO_2_^+^ redox reaction.

## Conclusions

To boost the economic appeal of the VRFB technology, enhancing and understanding the kinetics of vanadium redox reactions is extremely important to achieve high energy efficiency. HWO-C_76_ composites were fabricated and their electrocatalytic effects were investigated for the VO^2+^/VO_2_^+^ reaction. HWO showed a slight kinetics enhancement but a significant chlorine evolution inhibition in the mixed acid electrolyte. To further optimize the kinetics of HWO-based electrodes, different HWO:C_76_ ratios were used. Increasing the C_76_ content to HWO enhanced the electron transfer kinetics of the VO^2+^/VO_2_^+^ reaction on the modified electrode, with HWO-50% C_76_ being the optimum material as it decreased the charge transfer resistance and further inhibited chlorine evolution relative to C_76_ and TCC. This was ascribed to the synergistic effects between C=C sp^2^ hybridization, O–H, and W-OH functional groups. The degradation rate upon repetitive cycling for HWO-50% C_76_ was found to be 0.29 mV/cycle, compared to 0.33 mV/cycle and 0.49 mV/cycle for UCC and TCC, respectively, rendering it its high stability in the mixed acid electrolyte. The presented results successfully identified high-performance electrode materials for the VO^2+^/VO_2_^+^ reaction with fast kinetics and high stability. This would increase the output voltage and hence the energy efficiency of VRFBs, thereby decreasing their cost for future commercialization.

## Supplementary Information

Supplementary Information.

## Data Availability

The datasets used and/or analyzed during the current study are available from the corresponding author on reasonable request.
