# Supplementary material for: Tungsten oxide/fullerene-based nanocomposites as electrocatalysts and parasitic reactions inhibitors for VO2+/VO2+ in mixed-acids
Source: Sci Rep. 2022 Aug 23;12:14348. doi: 10.1038/s41598-022-18561-6 (PMC9399084; doi:10.1038/s41598-022-18561-6)
Supplement: Supplementary file 1 — Supplementary Information. [file 41598_2022_18561_MOESM1_ESM.docx]

**Supporting Information**

**Tungsten Oxide/Fullerene-Based Nanocomposites as Electrocatalysts and Parasitic Reactions Inhibitors for VO^2+^/VO_2_^+^** **in Mixed-Acids**

Farah A. El Diwany^a,b^, Taher Al Najjar^a^, Nageh K. Allam*^b^, Ehab N. El Sawy*^a^

^a^ Department of Chemistry, School of Sciences and Engineering, The American University in Cairo, New Cairo 11835, Egypt. E-mail: [Ehab.elsawy@aucegypt.edu](mailto:Ehab.elsawy@aucegypt.edu)

^b^Energy Materials Laboratory, School of Sciences and Engineering, The American University in Cairo, New Cairo 11835, Egypt. E-mail: [Nageh.allam@aucegypt.edu](mailto:Nageh.allam@aucegypt.edu)

**Equations:**

$I_{p}=\left( 2.99\times{10}^{5} \right)n^{\frac{3}{2}}AC(D\alpha)^{\frac{1}{2}}\nu^{\frac{1}{2}}$ (**S1**)

______________________________________________________________________________

$\Psi= \frac{-0.6288+0.0021( n\times{\Delta E}_{p})}{1-0.017(n\times{\Delta E}_{p})}$ for ΔE_p_ < 200 mV (**S2**)^1^

where all parameters have their known definitions.

______________________________________________________________________________

$\Psi=\frac{{(\frac{D_{o}}{D_{R}})}^{\frac{\alpha}{2}}k^{0}}{\sqrt{\frac{\pi D_{o}nF\nu}{\mathrm{RT}}}}$ (**S3**) ^1,2^

α was assumed to be 0.5 as it was found in Nicholson’s paper that the results were insensitive to the value of α. D_0_ and D_R_ are the diffusion coefficients of the oxidant and reductant species; D (V^4+^) = 4.22 x 10^-10^, D (V^5+^) = 4.03 x 10^-10^ m^2^ s^-1^.^3^

______________________________________________________________________________

$k^{0}=\frac{\mathrm{RT}}{n^{2}F^{2}AR_{\mathrm{CT}}C}$ (**S4**)^4^

**Tables:**

**Table S1:** Summary of ΔE_p_ and I_pc_/I_pa_ values of different carbon structures mentioned above for VO^2+^/ VO_2_^+^.

| Electrode material | Electrolyte | ν (mV s^-1^) | ΔE_p_ (mV) | I_pc_/I_pa_ | Ref. |
| --- | --- | --- | --- | --- | --- |
| GO nanoplatelets-GC | 2 M V^4+^/ 2 M H_2_SO_4_ | 10 | 230 | - | 5 |
| Mesoporous graphene-CF | 0.05 M V^3.5+^ / 1 M H_2_SO_4_ | 5 | 355 | 0.89 | 6 |
| N-mesoporous graphene-CF | 0.05 M V^3.5+^ / 1 M H_2_SO_4_ | 5 | 323 | 0.86 | 7 |
| CNFs-CF | 0.1 M V^4+^ / 2 M H_2_SO_4_ | 5 | 174 | - | 8 |
| CNFs-CNTs-CF | 0.1 M V^4+^/ 2 M H_2_SO_4_ | 5 | 136 | 0.84 | 9 |
| MWCNTs-CF | 0.1 M V^4+^/ 2 M H_2_SO_4_ | 2 | 252 | 0.85 | 10 |
| N-CNTs-CP | 0.1 M V V^4+^/ 2 M H_2_SO_4_ | 10 | 100 | - | 11 |
| Black pearl carbon-GC | 0.1 M V^4+^/ 2 M H_2_SO_4_ | 10 | 120 | 0.92 | 12 |
| Carbon sheets-CF | 0.1 M V^4+^/ 3 M H_2_SO_4_ | 10 | 355 | 0.74 | 13 |
| N-doped biomass-GC | 1.6 M V^4+^/ 3 M H_2_SO_4_ | 20 | 180 | 0.53 | 14 |
| Chitin-derived AC | 0.1 M V^4+^/ 0.1 M H_2_SO_4_ | 50 | 147 | - | 15 |
| Corn protein CB-CF | 0.1 M V^4+^/ 3 M H_2_SO_4_ | 5 | 146 | 0.87 | 16 |
| Cocoon-derived carbon | 1 M V^4+^/ 3 M H_2_SO_4_ | 5 | 166 | 0.8 | 17 |
| Wood-based carbon | 0.1 M V^4+^/ 3 M H_2_SO_4_ | 20 | 540 | - | 18 |
| GO-CNTs | 2 M V^4+^/ 2 M H_2_SO_4_ | 5 | 220 | - | 19 |
| Graphene-GF | 0.05 M V^4+^/ 1 M H_2_SO_4_ | 5 | 241 | 0.9 | 20 |
| N-graphene-GC | 1 M V^4+^/ 3 M H_2_SO_4_ | 50 | 100 | 0.72 | 21 |
| Carbon nanowalls | 0.5 M V^4+^/ 1 M H_2_SO_4_ | 5 | 190 | 0.83 | 22 |
| Ir-CNFs-GC | 1.6 M V^4+^/ 3 M H_2_SO_4_ | 20 | 170 | 0.64 | 23 |
| Carbon foam-GC | 1 M V^4+^/ 1 M H_2_SO_4_ | 100 | 286 | 0.85 | 24 |
| Corn protein C nanorods | 0.1 M V^4+^/ 2 M H_2_SO_4_ | 10 | 280 | - | 25 |
| C76- Untreated Carbon Cloth | 0.1 M V^4+^/1 M H_2_SO_4_+ 1 M HCl | 5 | 106 | 0.75 | 26 |

**Table S2:** Summary of ΔE_p_ values from the above studies on metal oxides towards VO^2+^/VO_2_^+^ redox reaction.

| Electrode material | Electrolyte | ν (mV s^-1^) | ΔE_p_ (mV) | Ref. |
| --- | --- | --- | --- | --- |
| CeO_2_-CF | 0.1 M V^4+^ / 2 M H_2_SO_4_ | 1 | 260 | 27 |
| ZrO_2_-CF | 0.1 M V^4+^ / 2 M H_2_SO_4_ | 1 | 224 | 28 |
| MoO_3_-CP | 1 M V^4+^ / 2.62 M H_2_SO_4_ | 10 | 130 | 29 |
| NiO-CF | 0.1 M V^4+^ / 3 M H_2_SO_4_ | 5 | 253 | 30 |
| SnO_2_-CF | 0.1 M V^4+^ / 3 M H_2_SO_4_ | 10 | 766 | 31 |
| Cr_2_O_3_-GF | 0.1 M V^4+^ / 3 M H_2_SO_4_ | 2.5 | 280 | 32 |

**Table S3:** Summary of the results from the CVs and Nyquist plots in Figures 5a and 5d, respectively, for the VO^2+^/VO_2_^+^ redox reaction.

|  | | **HWO** | | | **HWO-10% C_76_** | | | **HWO-30% C_76_** | | | **HWO-50% C_76_** | | | **C_76_** | | |
| --- | --- | --- | --- | --- | --- | --- | --- | --- | --- | --- | --- | --- | --- | --- | --- | --- |
| **VO^2+^/VO_2_^+^** | ν  (mV/s) | 5 | 20 | 50 | 5 | 20 | 50 | 5 | 20 | 50 | 5 | 20 | 50 | 5 | 20 | 50 |
|  | ΔE_p_ (mV) | 360 | 459 | 469 | 310 | 408 | 459 | 220 | 337 | 424 | 176 | 297 | 370 | 176 | 277 | 332 |
|  | I_pc_/I_pa_ | 0.46 | | | 0.51 | | | 0.64 | | | 0.71 | | | 0.82 | | |
|  | R_CT_(Ω) | 103 | | | 85 | | | 35 | | | 9 | | | 14 | | |
|  | W slope | -- | | | 0.9 | | | 1.04 | | | 1.32 | | | 0.94 | | |

**Table S4:** Nicholson’s parameter, Ψ, as obtained by Equations 2S and 3S. k^0^ values were calculated using slopes in Figure 5c, and Equation S4. k^0^ values from EIS data (Figure 5d) were obtained using Equation S5.

|  | | **HWO** | | | **HWO-10% C_76_** | | | **HWO-30% C_76_** | | | **HWO-50% C_76_** | | | **C_76_** | | |
| --- | --- | --- | --- | --- | --- | --- | --- | --- | --- | --- | --- | --- | --- | --- | --- | --- |
| **VO^2+^/VO_2_^+^** | ν (mV/s) | 5 | 20 | 50 | 5 | 20 | 50 | 5 | 20 | 50 | 5 | 20 | 50 | 5 | 20 | 50 |
|  | ΔE_p_ (mV) | 360 | 459 | 469 | 310 | 408 | 459 | 220 | 337 | 424 | 176 | 297 | 370 | 176 | 277 | 332 |
|  | Ψ | 0.026 | 0.01 | 0.01 | 0.043 | 0.02 | 0.01 | 0.102 | 0.03 | 0 | 0.13 | 0.05 | 0.02 | 0.132 | 0.1 | 0 |
|  | k^0^  (cm s^-1^)  **CV** | 0.421 x 10^-4^ | | | 0.774 x 10^-4^ | | | 2.08 x 10^-4^ | | | 2.5 x 10^-4^ | | | 2.28 x 10^-4^ | | |
|  | k^0^  (cm s^-1^)  **EIS** | 0.216 x 10^-4^ | | | 0.26 x 10^-4^ | | | 0.627 x 10^-4^ | | | 2.47 x 10^-4^ | | | 1.58 x 10^-4^ | | |

**Figures:**

**
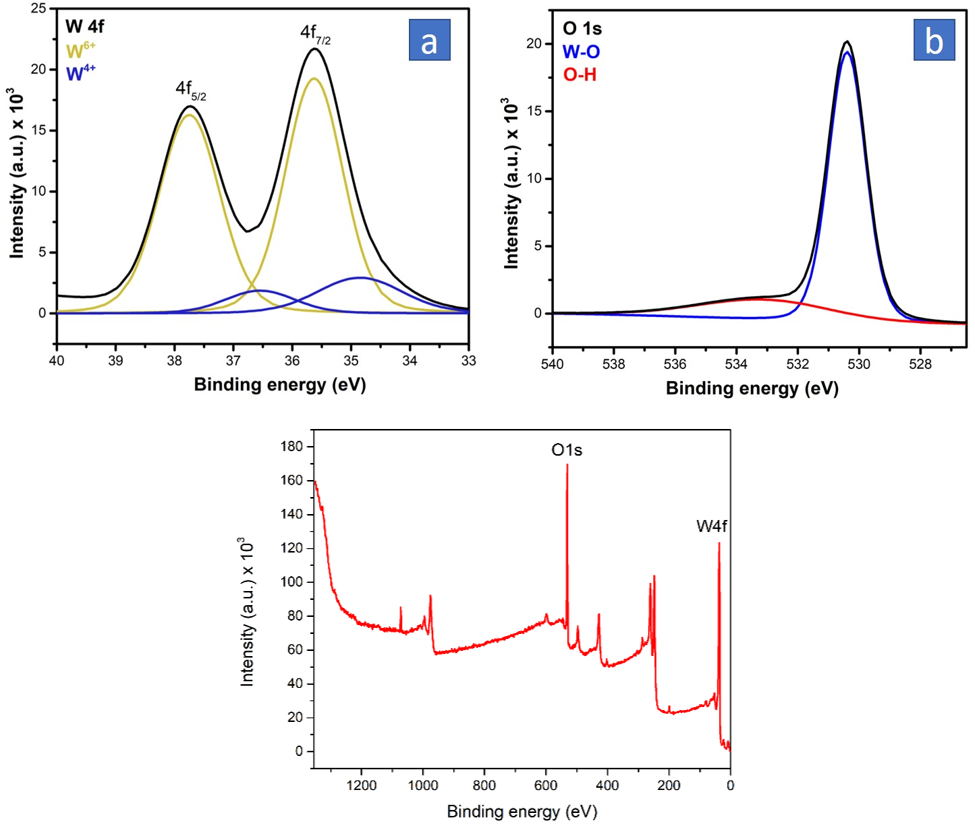
**

**Figure 1S:** Wide-range XPS scan spectrum of the synthesized hydrated tungsten oxide (HWO).

**Figure 2S:** The cyclic voltammetry behavior (at ν = 5 mV s-1 ) of the HWO-C76 composites with different HWO:C_76_ ratios for VO^2+^/VO_2_^+^ reactions in 0.1 M VOSO_4_/1 M H_2_SO_4_ + 1 M HCl electrolyte.

**References:**

1. Nicholson, R. S. & Nicholso.Rs. Theory and Application of Cyclic Voltammetry for Measurement of Electrode Reaction Kinetics. *Analytical Chemistry* **37**, 1351–1355 (1965).

2. Lavagnini, I., Antiochia, R. & Magno, F. An extended method for the practical evaluation of the standard rate constant from cyclic voltammetric data. *Electroanalysis* **16**, 505–506 (2004).

3. Jiang, Z., Klyukin, K. & Alexandrov, V. Structure, hydrolysis, and diffusion of aqueous vanadium ions from Car-Parrinello molecular dynamics. *Journal of Chemical Physics* **145**, 114303 (2016).

4. Wang, H. *et al.* Redox Flow Batteries: How to Determine Electrochemical Kinetic Parameters. *ACS Nano* **14**, 2575–2584 (2020).

5. Han, P. *et al.* Graphene oxide nanoplatelets as excellent electrochemical active materials for VO2+/VO2+ and V2+/V3+ redox couples for a vanadium redox flow battery. *Carbon* **49**, 693–700 (2011).

6. Opar, D. O., Nankya, R., Lee, J. & Jung, H. Three-dimensional mesoporous graphene-modified carbon felt for high-performance vanadium redox flow batteries. *Electrochimica Acta* **330**, 135276 (2020).

7. Opar, D. O., Nankya, R., Lee, J. & Jung, H. Assessment of three-dimensional nitrogen-doped mesoporous graphene functionalized carbon felt electrodes for high-performance all vanadium redox flow batteries. *Applied Surface Science* **531**, 147391 (2020).

8. Wei, G., Fan, X., Liu, J. & Yan, C. Investigation of the electrospun carbon web as the catalyst layer for vanadium redox flow battery. *Journal of Power Sources* **270**, 634–645 (2014).

9. Park, M., Jung, Y. J., Kim, J., Lee, H. Il & Cho, J. Synergistic effect of carbon nanofiber/nanotube composite catalyst on carbon felt electrode for high-performance all-vanadium redox flow battery. *Nano Letters* **13**, 4833–4839 (2013).

10. Wei, G., Jia, C., Liu, J. & Yan, C. Carbon felt supported carbon nanotubes catalysts composite electrode for vanadium redox flow battery application. *Journal of Power Sources* **220**, 185–192 (2012).

11. Sodiq, A. *et al.* Enhanced electrochemical performance of modified thin carbon electrodes for all-vanadium redox flow batteries. *Materials Advances* **1**, 2033–2042 (2020).

12. Sodiq, A. *et al.* Black pearl carbon as a catalyst for all-vanadium redox flow batteries. *Chemical Communications* **55**, 10249–10252 (2019).

13. Gao, Y. *et al.* Carbon sheet-decorated graphite felt electrode with high catalytic activity for vanadium redox flow batteries. *Carbon* **148**, 9–15 (2019).

14. Cheng, D. *et al.* One-step activation of high-graphitization N-doped porous biomass carbon as advanced catalyst for vanadium redox flow battery. *Journal of Colloid and Interface Science* **572**, 216–226 (2020).

15. Wan, C. T. C. *et al.* Exploration of Biomass-Derived Activated Carbons for Use in Vanadium Redox Flow Batteries. *ACS Sustainable Chemistry and Engineering* **8**, 9472–9482 (2020).

16. Park, M., Ryu, J., Kim, Y. & Cho, J. Corn protein-derived nitrogen-doped carbon materials with oxygen-rich functional groups: A highly efficient electrocatalyst for all-vanadium redox flow batteries. *Energy & Environmental Science* **7**, 3727–3735 (2014).

17. Wang, R. & Li, Y. Twin-cocoon-derived self-standing nitrogen-oxygen-rich monolithic carbon material as the cost-effective electrode for redox flow batteries. *Journal of Power Sources* **421**, 139–146 (2019).

18. Jiao, M. *et al.* Holey three-dimensional wood-based electrode for vanadium flow batteries. *Energy Storage Materials* **27**, 327–332 (2020).

19. Han, P. *et al.* Graphene oxide nanosheets/multi-walled carbon nanotubes hybrid as an excellent electrocatalytic material towards VO 2+/VO 2+ redox couples for vanadium redox flow batteries. *Energy & Environmental Science* **4**, 4710–4717 (2011).

20. González, Z. *et al.* Outstanding electrochemical performance of a graphene-modified graphite felt for vanadium redox flow battery application. *Journal of Power Sources* **338**, 155–162 (2017).

21. Jin, J. *et al.* Identifying the Active Site in Nitrogen-Doped Graphene for the VO2+/VO2+ Redox Reaction. *ACS Nano* **7**, 4764–4773 (2013).

22. González, Z., Vizireanu, S., Dinescu, G., Blanco, C. & Santamaría, R. Carbon nanowalls thin films as nanostructured electrode materials in vanadium redox flow batteries. *Nano Energy* **1**, 833–839 (2012).

23. Cheng, D. *et al.* Endowing electrospun carbon fiber with excellent electrocatalytic properties towards VO2+/VO2+ redox reaction for vanadium redox flow battery by in situ iridium decoration. *Colloids and Surfaces A: Physicochemical and Engineering Aspects* **586**, 124137 (2020).

24. Jeong, S., An, S., Jeong, J., Lee, J. & Kwon, Y. Effect of mesocelluar carbon foam electrode material on performance of vanadium redox flow battery. *Journal of Power Sources* **278**, 245–254 (2015).

25. Aziz, M. A., Hossain, S. I. & Shanmugam, S. Hierarchical oxygen rich-carbon nanorods: Efficient and durable electrode for all-vanadium redox flow batteries. *Journal of Power Sources* **445**, 227329 (2020).

26. Diwany, F. A. E., Ali, B. A., Sawy, E. N. E. & Allam, N. K. Fullerene C76 as a novel electrocatalyst for VO2+/VO2+ and chlorine evolution inhibitor in all-vanadium redox flow batteries. *Chem. Commun.* **56**, 7569–7572 (2020).

27. Zhou, H. *et al.* CeO2 decorated graphite felt as a high-performance electrode for vanadium redox flow batteries. *RSC Advances* **4**, 61912–61918 (2014).

28. Zhou, H., Shen, Y., Xi, J., Qiu, X. & Chen, L. ZrO2-Nanoparticle-Modified Graphite Felt: Bifunctional Effects on Vanadium Flow Batteries. *ACS Applied Materials and Interfaces* **8**, 15369–15378 (2016).

29. Cao, L., Skyllas-Kazacos, M. & Wang, D. W. Modification Based on MoO3 as Electrocatalysts for High Power Density Vanadium Redox Flow Batteries. *ChemElectroChem* **4**, 1836–1839 (2017).

30. Yun, N., Park, J. J., Park, O. O., Lee, K. B. & Yang, J. H. Electrocatalytic effect of NiO nanoparticles evenly distributed on a graphite felt electrode for vanadium redox flow batteries. *Electrochimica Acta* **278**, 226–235 (2018).

31. Mehboob, S. *et al.* Enhancing the performance of all-vanadium redox flow batteries by decorating carbon felt electrodes with SnO2 nanoparticles. *Applied Energy* **229**, 910–921 (2018).

32. Xiang, Y. & Daoud, W. A. Cr2O3-modified graphite felt as a novel positive electrode for vanadium redox flow battery. *Electrochimica Acta* **290**, 176–184 (2018).
